# The Protective Effect of Stauntonia Chinensis Polysaccharide on CCl_4_-induced Acute Liver Injuries in Mice

**Published:** 2014-03

**Authors:** Jiaojiao Yang, Qingming Xiong, Jing Zhang, Shirong Yan, Lihua Zhu, Bo Zhu

**Affiliations:** 1 First Clinical College, Hubei University of Medicine, Shiyan, Hubei, China;; 2 School of Basic Medical Sciences, Hubei University of Medicine, Shiyan, Hubei, China

**Keywords:** Stauntonia chinensis polysaccharides, Liver injury, Antioxidation

## Abstract

**Objective:**

To investigate the protective effect of Stauntonia chinensis polysaccharides (*SCP*) on carbon tetrachloride (CCl_4_) induced acute liver injuries in mice.

**Methods:**

Kunming mice were randomly divided into three groups: the control group, the pathological model group, and the *SCP* group. The *SCP* group was further divided into three subgroups based on *SCP* treatment: Low dosage (50 mg/kg), medium dosage (100 mg/kg) and high dosage (200 mg/kg). After being fed for 7 days, mixed-edible-oil solution was intraperitoneally injected into the control group, while the other two groups were injected with 0.15% CCl_4_ mixed with mixed-edible-oil. Sera were collected from mice 24 h later to determine the activity of serum alanine transaminase (ALT). Mice were then sacrificed to prepare liver homogenate. Levels of liver malondialdehyde (MDA), glutathione (GSH), glutathione peroxidase (GSH-Px), superoxide dismutase (SOD) and nitric oxide (NO) were determined. Pathological changes in livers were also analyzed.

**Results:**

*SCP* significantly reduced the ALT activity in the serum and inhibited the decrease of serum GSH, GSH-PX and SOD and rose the levels of MDA and NO (*P*<0.05-0.01). This lessened the pathological damage to the liver tissues.

**Conclusion:**

*SCP* protects against CCl_4_-induced acute liver injuries in mice.

## INTRODUCTION

Stauntonia chinensis polysaccharides (*SCP*) is an important active compound extracted from an edible medicinal plant Stauntonia Chinensis. Previous studies have shown that *SCP* is able to scavenge oxygen free radicals and exhibit a significant antioxidant activity (1). Stauntonia chinensis has been shown to reduce hepatocellular steatosis and necrosis induced by the subcutaneous injection of carbon tetrachloride (CCl_4_) and to stimulate the repairing of liver cells (2). However, what kind of material plays a protective role in this process is still unknow. So in this study, we examined the hypothesis that *SCP* may have the ability to reduce the acute liver injuries induced by CCl_4_. CCl_4_ is a chemical toxin that induces liver injury (3, 4) via oxidative stress (5). Therefore, 0.15% CCl_4_ was used to induce acute liver injuries in mice to explore the protective effect of *SCP* and we are expecting to provide the experimental evidence for the protecting effect of *SCP*.

## MATERIALS AND METHODS

### Drugs & Reagents

Fifty Kunming mice aged 4 months were randomly chosen with weighing between 32 and 38 g which happened to be equal in number in gender were fed separately in cages. Animals were housed in the medical school animal center with Animal Qualification Certificate No. SCXK (Hubei) 20110008. ALT, AST, MDA, GSH, GSH-PX, SOD, NO and protein test kits were purchased from Nanjing Jiancheng Bioengineering Institute, the pawpaw from Shiyan Guoren Drugstore, and all other analytical reagents were made in China.

### Equipments

RA-50 Semiauto Biochemical Analyzer (Technlcon, U.S).

### Extraction of *SCP*

Dried pawpaw powder was diluted with double distilled water at a ratio of 1:15. The mixture was incubated at 80°C for 6 h, filtered through a 4-layer gauze, and cooled. The supernatant was centrifuged at 4000 rpm for 10 min to remove impurities and concentrated at 90°C. The *SCP* precipitated when the ethanol concentration reached 80%. Once the *SCP* precipitated out, it was collected after centrifugation at 4000 rpm for 10 min. Ethanol was removed, and the sediments were extracted three times by anhydrous ethanol (6). Extracts were then vacuum-dried to obtain brown *SCP* at 23.8% content, as determined by anthrone colorimetry (7).

### Method

Fifty mice were randomly divided into control group, pathological model group, low *SCP* group, medium *SCP* group, and high *SCP* group, with each 10 mice per group. The control and pathological model groups were lavaged once every day with 0.2 ml saline; the low, medium, and high *SCP* groups were lavaged once every day with 50, 100, and 200 mg/kg *SCP* in 0.2 ml, respectively. Mice were fed continually for 7 day. Two hours after the last feeding, the control group was given intraperitoneal injection of mixed-edible-oil solution (10 ml/kg), while the rest of the groups received intraperitoneal injections of 0.15% CCl_4_ and mixed-edible-oil solution. No food intake was allowed in the following 12 h except water. Twenty-four hours later, intraorbital blood was taken. Serum ALT concentration was determined with Reitman-Frankel method. Mice were euthanized and about 1 cm × 1 cm × 0.5 cm tissue of the right lobe of the liver was dissected. The liver tissue was fixed with 10% formalin, HE-stained, and analyzed for the degree of liver injury. The rest of the liver was prepared for a liver homogenate. After centrifugation, protein content in the supernatant was determined with the Biuret method. The MDA, SOD, GSH, GSH-PX and NO concentrations in tissues and sera were determined according to manufacturer’s protocols of corresponding kits.

### Statistical Analysis

Data were analyzed with SPSS 13.0 software. Results were shown in x ± s. The mean comparison among groups was performed using the single factor variance analysis. Statistical significance was defined as *P*<0.05.

## RESULTS

### The Effect of *SCP* on ALT and AST in Serum

Reitman-Frankel method was used to determine the serum ALT concentration. Also the MDA and SOD concentrations in tissues and sera were determined according to manufacturer’s protocols of corresponding kits. The concentrations of ALT (30.02 vs 183) and MDA (1.25 vs 1.67) in the pathological model group was significantly rose compared with the control group (*P*<0.05-0.01), indicating that the model is successful. The activities of ALT and MDA were declined compared with the model group, together with the activity of SOD was increased, which is an indication that the injuries was attenuated. However, there weren’t correlation among there subgroups (date not shown). In addition, the activity of SOD did not differ between low *SCP* group and model group. These results showed that *SCP* protective effect may be not does-depended (Table 1).

**Table 1:** The Effect of *SCP* on ALT and AST in Serum

Groups	ALT/(U/L)	MDA/(nmol/mg protein)	SOD/(U/mg protein)

Control	30.02 ± 2.76^b^	1.25 ± 0.11^a^	154.78 ± 9.48^a^
Model	183 ± 53.21	1.67 ± 0.17	139.98 ± 10.15
low *SCP* group	130.79 ± 20.90^a^	0.83 ± 0.07^b^	147.14 ± 19.15
medium *SCP* group	128.22 ± 21.36^a^	0.98 ± 0.25^a^	156.23 ± 10.82^a^
high *SCP* group	106.02 ± 35.44^a^	0.78 ± 0.12^a^	166.41 ± 21.54^a^

Data are presented as mean ± SEM (n=10). Significant differences with the model group were designated as

a *p*<0.05.

b *p*<0.01.

### The Effect of *SCP* on GSH, GSH-PX and NO with Liver Injuries Caused by CCI_4_ in Mice

The GSH, GSH-PX and NO concentrations in tissues and sera were determined according to manufacturer’s protocols of corresponding kits. The results were similar with above. The changes of GSH, GSH-PX and NO were significantly in contrast to the model group (*P*<0.05-0.01). It may be concerned with that *SCP* neutralizes and scavenges the free radicals, thereby inhibiting lipid per-oxidative reaction. The activities of GSH and GSH-PX in three *SCP* groups were all increased compared with the model group; this indicated that *SCP* could attenuate the per-oxidative injury induced by CCl_4_. But there were also no differences in effects among the three subgroups (date not shown) (Table 2).

**Table 2:** The Effect of *SCP* on GSH, GSH-PX and NO

Groups	GSH/(mg/g protein)	GSH-PX/(U/mg protein)	N0/(μmol/g protein)

control	1.30 ± 0.18^b^	935.93 ± 17.35^b^	2.53 ± 0.02^b^
model	0.90 ± 0.10	876.73 ± 29.80	5.54 ± 0.42
low *SCP* group	1.13 ± 0.13^b^	925.07 ± 42.79^a^	4.15 ± 0.29^b^
medium *SCP* group	1.15 ± 0.08^b^	936.34 ± 38.89^b^	2.94 ± 0.34^b^
high *SCP* group	1.28 ± 0.12^b^	925.07 ± 30.89^b^	2.64 ± 0.85^b^

Data are presented as mean ± SEM (n=10). Significant differences with the model group were designated as

a *p*<0.05.

b *p*<0.01.

### The Effect of *SCP* on Pathological Changes of Liver Tissue Caused by CCI_4_

**Figure 1:**
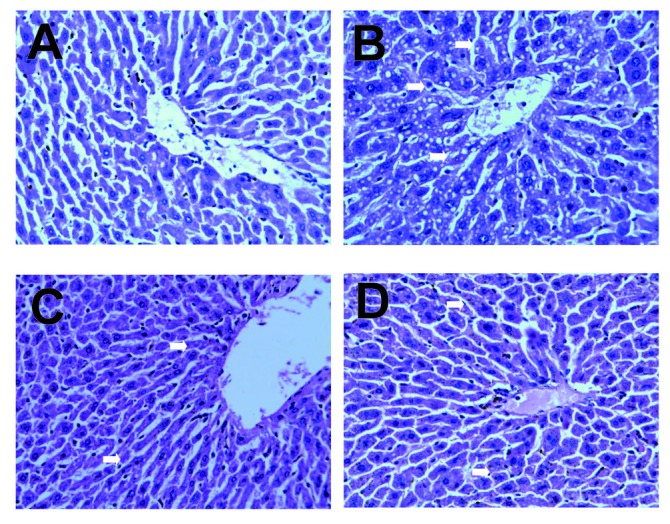
Pathological Sections of Mice Liver Tissue (HE-stained ×400). **A,** Control group; **B,** Model group: Medium to severe steatosis, spotty hepatocellular necrosis and lymphocyte infiltration in portal area; **C,** Medium *SCP* group: light steatosis and spotty necrosis; **D,** High *SCP* group: light steatosis without obvious necrosis seen.

We observed the pathological changes of liver tissues by HE staining. In the control group, liver tissues radiated around the central veins, and liver cells were arranged neatly without degeneration, steatosis or necrosis. In the model group, spotty necrosis was observed mainly around the central vein of the hepatic lobule. We also observed medium to severe steatosis in the liver lobule and lymphocyte infiltration in portal area, showing obvious injuries in the liver tissue. Compared with the model group, liver tissue injuries in medium and high *SCP* groups were less serious by varying extents. Spotty necrosis was reduced, and steatosis and interstitial inflammation were not as severe as the model group indicated that* SCP* can protect against hepatocellular oxidative injury in mice. Yet the difference between them was not obvious, which was consistent with the results above mentioned (Figure 1).

## DISCUSSION

In this study, we investigated whether *SCP* could ameliorate liver damage and our results clearly revealed that *SCP* could attenuate liver damage, and this effect might not only be a prevention of hepatocellular necrosis, but also a promotion of recovery from liver injury. Polysaccharides have many biological activities, such as protecting against lipid per-oxidation, aging,and tumor growth. It can also remove free radicals, reduce blood lipids, enhance immunity, and protect animal liver from injuries (8-10). Tian and coworkers showed that Stauntonia chinensis had a protective effect on liver damage induced by CCl_4_ (2). Animal studies have shown that antioxidant enzyme levels depend on the availability of antioxidants in diet. It can be hypothesized that *SCP* may play the role.

Although oxidation is essential to many living organisms for the production of energy to fuel biological processes, oxidative stress can damage biological molecules. CCl_4_-induced liver injury is the result of a typical lipid per-oxidation damage. ALT, MDA, NO level increased and SOD, GSH, GSH-PX decreased significantly suggesting that CCl_4_ toxicity caused by the trichloromethyl radical is existed in the model group. When CCl_4_ enters the body and is metabolized by the hepatic microsomal cytochrome P450, trichloromethyl radical (CC1) and chloride ion radicals (C1) are generated. These radicals can be covalently bound to the macromolecules in liver cells and attack the phospholipid molecules on liver cell membrane. This causes lipid per-oxidation (11), which ultimately leads to the damage of liver cells and even necrosis. As a result, ALT is released from the injured liver cells, which can be detected by the rise in serum transaminase (12). MDA, the end product of lipid per-oxidation metabolism during the course of the radical attack on lipids, can further damage the liver cells. MDA concentration may indirectly reflect the degree of liver injury (13). In this study, the ALT and MDA activities in the *SCP* groups were significantly low when compared with model group in Table 1. NO inhibits hepatocellular protein synthesis, disrupts DNA structure, inhibits mitochondrial oxidative respiratory function, induces energy metabolism in the liver cells, and induces liver cell apoptosis and necrosis. NO reacts with oxygen free radicals and initiates lipid per-oxidative chain reaction to amplify the effect of free radical injury. This is one of the main free radicals responsible for oxidative stress injury. As seen in Table 2, in the *SCP* groups the levels of liver NO was significantly lower than in the model group. The decreased ALT, MDA and NO levels in the liver indicate that the production of oxygen-free radicals was effectively inhibited by *SCP*.

SOD catalyzes the free radical for disproportionating reaction to prevent the secondary reaction between free radicals and membrane lipids and proteins. This protects against damage to the liver cells (14). SOD levels were significantly higher in the *SCP* groups compared with model group in Table 1. GSH and GSH-PX, an important peroxide catabolic enzyme in the body, remove free radicals and protect cell membrane and protein from injuries induced by free radicals. There was an increase in the levels of GSH-PX and GST in liver of the *SCP* groups compared with model group, indicating *SCP* also be a promotion of recovery from liver injury (Table 2).

Parallel with the date results, our pathological sections showed that steatosis and necrosis were seriously in model group compared with the control group, which indicated that our model was successful. In medium and high *SCP* groups, necrosis was reduced and steatosis was attenuated. This further indentified that *SCP* could protect against hepatocellular oxidative injury in mice.

These studies have provided some evidence for *SCP* in the prevention of CCl_4_-induced acute liver injuries in mice. We conclude that *SCP* protects against liver injuries in mice. The underlying mechanism may be *SCP* can neutralize and scavenge the free radicals, thereby inhibiting lipid per-oxidative reaction and promote liver regeneration and repair. However, the anti-oxidation effect among the *SCP* groups has some differences but was not significant. We are planning further studies to investigate the protective effect of SCP with other toxins, factor or infection to extend our understanding of the issues raised by our results.
